# Molecular mechanism of crisaborole combined with erythromycin against methicillin-resistant *Staphylococcus aureus in vivo* and *in vitro*

**DOI:** 10.3389/fmicb.2025.1503515

**Published:** 2025-02-27

**Authors:** Yinuo Zou, Lei Yu, Jinjing Xue, Tianqi Fang, Liping Sun, Jianfeng Wang, Minhe Cui, Peng Zhang, Yonglin Zhou

**Affiliations:** ^1^Thoracic Surgery Department, The First Hospital of Jilin University, Changchun, Jilin, China; ^2^State Key Laboratory for Diagnosis and Treatment of Severe Zoonotic Infectious Diseases, Key Laboratory for Zoonosis Research of the Ministry of Education, Institute of Zoonosis, and College of Veterinary Medicine, Jilin University, Changchun, China; ^3^Department of Physiology, Basic Medical College, Jilin University, Changchun, Jilin, China; ^4^Key Laboratory of Ministry of Education for Conservation and Utilization of Special Biological Resources in the Western China, and School of Life Sciences, Ningxia University, Yinchuan, China; ^5^Affiliated Hospital of Changchun University of Traditional Chinese Medicine, Changchun, Jilin, China; ^6^Jilin Mushuo Breeding Co., Ltd, Changchun, Jilin, China

**Keywords:** crisaborole, erythromycin, methicillin-resistant *Staphylococcus aureus*, synergistic effect, skin infection

## Abstract

The widespread presence of methicillin-resistant *Staphylococcus aureus* (MRSA) severely threatens convenient therapeutic options in the postantibiotic era. The use of combinations of existing drugs at this stage may be a viable strategy for dealing with complex drug-resistant MRSA infections. An checkerboard MIC (Minimum Inhibitory Concentration) assay, growth curve assay, bactericidal test and scanning electron microscope (SEM) assays were performed to determine whether crisaborole (AN2728), a PDE4 inhibitor for treating atopic dermatitis (AD), produced bactericidal effect with different antibiotics. Here, we identified (AN2728) produced a significant synergistic bactericidal effect with erythromycin, cefuroxime and rifampicin against different bacterial strains of *Staphylococcus aureus* (*S. aureus*), especially MRSA (FIC < 0.5) (*p* < 0.05). Transcriptome analysis, bacterial biofilm assay and several kit assays revealed that AN2728 could also simultaneously affect the membrane and transporter capacity of MRSA. Moreover, in a mouse skin infection model of MRSA, the combination of AN2728 and erythromycin showed remarkable treatment benefits, as shown by significantly reduced bacterial loading (*p* < 0.05), pathological lesions of the skin and an obvious anti-inflammatory effect (*p* < 0.05). To our knowledge, this study is the first to establish that AN2728 can cooperate with antibiotics such as erythromycin to completely kill MRSA and that AN2728 can be used to extend the usage life of different antibiotics to address the inevitability of severe MRSA infection.

## Graphical abstract

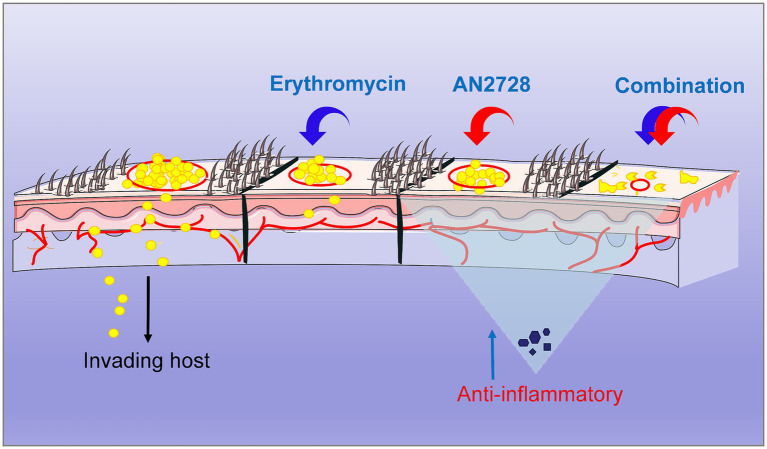

## Introduction

*Staphylococcus aureus* (*S. aureus*) remains the most problematic and costly source of gram-positive bacterial infection worldwide and has been a cause for global public concern for decades (Lowy, 1998). Clinically, a core issue associated with *S. aureus* is the remarkable level of resistance to different conventional antibiotics, complicating treatment, with methicillin-resistant *S. aureus* (MRSA) predominating (Guo et al., 2020). Therefore, the failure of antimicrobial therapy for skin and soft tissue infections, severe endocarditis, bacteremia, necrotizing pneumonia, bone and joint infections and hospital-acquired infections may be mainly due to the presence of MRSA. The prevention and control of MRSA infection is still an important research focus (Kramer et al., 2019). Factors such as global economic recession and increased warfare have slowed the development of new antibiotics, and new strategies to combat MRSA infections are urgently needed. The new use of old drugs or the discovery of new pharmacological agents for commercial drugs is an effective strategy for addressing the difficulties associated with treating MRSA.

Erythromycin is currently the most commonly used macrolide antibiotic derived from *Streptomyces erythreus*. The mechanism of action of erythromycin is fundamentally the inhibition of protein synthesis by binding 23S ribosomal RNA of the 50S ribosomal subunit. It has a broad-spectrum antimicrobial effect against gram-positive organisms and can be prepared in oral formulations, ointments and injections (Hobson, 1954; Brittain, 1987). However, *S. aureus* independently acquired the SCC*mec* complex during its evolution in the early 1960s, resulting in resistance to erythromycin, penicillin, streptomycin, and tetracycline (Crisóstomo et al., 2001). It has long been known that erythromycin-resistant *Staphylococci* can be expected to be at least as common as those resistant to antibiotics (Bradley, 2005). Nevertheless, erythromycin in combination with other types of antibiotics still has a good anti-infective effect, and erythromycin is prepared as an ointment for the treatment of skin infections and soft tissue infections caused by different gram-positive bacteria in many countries (Sun et al., 2021; National Institute of Child Health and Human Development, 2019). Typically, erythromycin can be potentiated with penicillins, cephalosporins, and tetracyclines. Therefore, the combination of erythromycin with other drugs for the treatment of MRSA infection is still a very promising strategy (Adamantidi et al., 2024).

Crisaborole (AN2728), a nonsteroidal phosphodiesterase 4 (PDE4) inhibitor, represents the first medication approved by the FDA for the treatment of different skin diseases, including atopic dermatitis (AD) and off-label treatment for recalcitrant palmoplantar psoriasis, inflammatory linear verrucous epidermal nevus, seborrheic dermatitis and vitiligo (Makins et al., 2020; Robbins et al., 2018). AN2728 is a versatile boron-heterocyclic scaffold with a wide range of physicochemical and drug-like properties, which has led to the discovery of new applications in medicinal chemistry research (Yang et al., 2003; Rock et al., 2007; Ciani and Ristori, 2012; Zhang et al., 2013). Structural modification of AN2728 has rendered it antibacterial, antifungal, antiparasitic and antiviral (Rock et al., 2007; Ciani and Ristori, 2012). Indeed, *S. aureus* is one of the leading causes of AD. Nevertheless, whether AN2728 alone or in combination with erythromycin can treat bacterial dermatitis has not been reported. Here, we investigated the therapeutic efficacy of AN2728 in combination with erythromycin against *S. aureus* skin infections, and the therapeutic efficacy of AN2728 in combination with other antibiotics needs to be further explored (mostly synergistic or additive). Thus, our study provides a potential strategy for combining new and old drugs to completely eradicate bacterial skin and soft tissue infections and prevent dermatitis.

## Materials and methods

### Bacterial strains and chemical reagents

The bacterial strains of *S. aureus* used in this study included *S. aureus* ATCC25904, *S. aureus* ATCC29213, *S. aureus* ATCC25923, MRSA USA300, MRSA USA400 and MRSA 252, which were obtained from the American Type Culture Collection (ATCC), and MRSA ST144, which was obtained from Professor Kui Zhu of China Agricultural University.

AN2728 was purchased from Chengdu Derick Biotechnology Co., Ltd. (Chengdu, China). All antibiotics were purchased from the National Institute for the Control of Pharmaceutical and Biological Products (Beijing, China) and Shanghai Yuanye Biotechnology Co., Ltd. (Shanghai, China). Erythromycin ointment was purchased from Xinxiang Huaqing Pharmaceutical Co., Ltd. (Xinxiang, China). Unless otherwise stated, all chemical reagents were purchased from Sigma–Aldrich, Inc. (St. Louis MO, USA) (content of the main component ≥99.7%).

### Growth curve assay

For the growth curve assay, overnight cultured MRSA USA300 was cultured at 37°C to obtain a starting OD_600nm_ = 0.3. The cultures were divided into six 50 mL sterilized Erlenmeyer flasks, and AN2728 was added to the cultures at final concentrations of 0, 2, 4, 8, 16, and 32 μg/mL. Then, the bacteria in each Erlenmeyer flask were cultured with shaking (220 rpm) at 37°C, and growth was continuously detected by measuring the OD_600 nm_ value with a UV spectrophotometer every 30 min.

### Checkerboard microdilution assay

The synergistic activity between AN2728 and different antibiotics against bacterial strains of *S. aureus* was identified by checkerboard MIC assays according to the Clinical and Laboratory Standards Institute (CLSI) protocol (Clinical and Laboratory Standards Institute, 2015). Briefly, the tested antibiotic was serially diluted twofold with 100 μL of MHB along the abscissa, and each column of AN2728 was serially diluted twofold, which contained approximately 5 × 10^5^ colony forming units (CFUs) mL^−1^ of bacterial suspensions. After 18–24 h of incubation at 37°C, the MIC values were defined as the lowest concentrations of antibiotics with no visible growth of bacteria. The fractional inhibitory concentration (FIC) index values were calculated by the following formula:


$$\begin{array}{l} FIC\;\mathrm{index}={\mathrm{MIC}}_{\mathrm{AN}2728\;\mathrm{alone}}/{\mathrm{MIC}}_{\mathrm{AN}2728\;\mathrm{in combination}}+\\ {\mathrm{MIC}}_{\mathrm{antibiotics alone}}/{\mathrm{MIC}}_{\mathrm{antibiotics in combination}} \end{array}$$

The synergistic effect was defined as an FIC < 0.5 (Clinical and Laboratory Standards Institute, 2015). All MICs were determined with three biological replicates.

### Bactericidal test

Time-dependent killing curve and plating sterilization tests were used to confirm the bactericidal effect of AN2728 and erythromycin (Xu et al., 2022). Mid-logarithmic-phase bacteria of MRSA USA300 were separately diluted to 5 × 10^5^ CFU/mL in each well of a sterilized 96-well microlitre plate, and AN2728 (1/4 × MIC), erythromycin (1/4 × MIC) and AN2728 (1/4 × MIC) combined with erythromycin (1/4 × MIC) were added. Then, the bacterial cultures in each well were incubated at 37°C and removed at 0–24 h postinoculation for bacterial counts. Ten microliters of serial 10-fold dilutions were spotted on TSB agar plates, and the number of bacterial colonies was determined after overnight incubation at 37°C.

### Combined disk test

MRSA USA300 strains cultured overnight or diluted were placed on Luria Broth agar plates. Five microliters of various concentrations of AN2728 (0, 4 and 8 μg) were added to erythromycin discs (0, 15 and 30 μg). The inhibition zone diameters around the erythromycin disks were compared after 12 h, 24 h, and 36 h of incubation at 37°C.

### Bacterial biofilm assay

Overnight-cultured and diluted MRSA USA300 was cultured in TSB broth in sterilized 24-well microliter plates with different concentrations of AN2728. After 6 h of incubation, 12 h of incubation, and 24 h of incubation, the membrane from which the MRSA USA300 formed was dyed with crystal violet (CV), and the OD_570 nm_ of the membrane was measured with a microplate reader.

### Live/dead bacteria staining

The live/dead status of the bacteria subjected to different treatments was visually observed by live/dead bacteria staining. At the mid-logarithmic phase, MRSA USA300 was diluted in TSB broth supplemented with AN2728 (4 μg/mL), erythromycin (8 μg/mL) or the combination of both for 5 h at 37°C. Then, the bacterial cultures were collected by centrifugation at 5000 rpm for 10 min, and sterile PBS buffer supplemented with fluorochrome (LIVE/DEAD BacLight Bacterial Viability Kit, Thermo Fisher Scientific, Shanghai, China) was used to resuspend the bacterial cultures. Red fluorescence and green fluorescence indicate dead bacteria and live bacteria, respectively.

### Scanning electron microscope (SEM) assays

The tested MRSA USA300 strains were proportionally diluted in TSB broth supplemented with AN2728 (4 μg/mL), erythromycin (8 μg/mL) or the combination of both and cultured for 5 h at 37°C. Then, the bacterial cultures were collected by centrifugation, washed with PBS and resuspended in PBS to obtain an OD_600nm_ of 0.5. The bacterial samples were fixed in 4% glutaraldehyde overnight at 4°C and further fixed with osmium acid for 2 h at 4°C. Then, the samples were gradually dehydrated with ethanol, dried with a carbon dioxide critical point dryer, and sprayed with gold. Finally, the morphological changes of the tested bacteria were observed by using a scanning electron microscope (HITACHI SU8010, GeminiSEM300, Germany).

### Transcriptome analysis

Overnight-cultured MRSA USA300 was proportionally diluted in TSB broth with or without AN2728 (4 μg/mL) and cultured for 8 h at 37°C. Then, the bacterial samples were washed with fresh PBS buffer and collected by centrifugation at 12000 rpm for 10 min at 4°C. The construction and sequencing of cDNA libraries and transcriptome data collection were performed by Beijing Novogene Technology Co., Ltd. (Beijing, China) (Zhou et al., 2022). And we have uploaded the data into the NCBI library (PRJNA1092827).

### Intracellular ATP determination

Overnight cultured MRSA USA300 strains were diluted 1:100 in TSB broth, and different concentrations of AN2728 (0 or 2–32 μg/mL) were added to the cultures at 37°C for 5 h. The MRSA USA300 bacterial samples were collected, washed, and resuspended in PBS. Then, the intracellular ATP levels were detected using an Enhanced ATP Assay Kit (Beyotime, Shanghai, China) according to the manufacturer’s protocol (Song et al., 2021).

### Inner membrane (IM) integrity determination

The bacterial samples of MRSA USA300 were collected, washed, resuspended and incubated with different concentrations of AN2728 for 1 h. Then, 5 μM propidium iodide (PI; Sigma–Aldrich, St. Louis, MO, USA) was added for coincubation at 37°C for 30 min, and the membrane integrity was determined with an excitation wavelength of 535 nm and an emission wavelength of 615 nm using an Infinite M200 microplate reader (TECAN, Switzerland).

### Membrane depolarization analysis

Bacterial samples were collected, washed and adjusted to an OD_600_ of 0.5. 3,3-Dippingropylthiadicarbocyanine iodide DiSC_3_(5) (Aladdin, Shanghai, China) was added to the samples for 10 min. Then, the samples were supplemented with different concentrations of AN2728 for an additional 30 min. Membrane depolarization was measured with an excitation wavelength of 622 nm and an emission wavelength of 670 nm.

### Mouse skin infection assays

Six- to eight-week-old female BALB/c mice were obtained from Liaoning Changsheng Biotechnology Co., Ltd. The animal experiments were approved and conducted following the guidelines of the Animal Welfare and Research Ethics Committee at Jilin University.

The mice (*n* = 6 per group) were deprived of dorsal hair, anesthetized and perforated on the back using a perforator. Drops of 20 μL (1 × 10^7^ CFU) of resuspended MRSA USA300 were placed in the wound 3 times consecutively to establish an *S. aureus-induced* septic skin infection model. After successful infection establishment, the mice were epidermically administered erythromycin ointment (5 mg), AN2728 ointment (50 mg, 2%), and erythromycin ointment (5 mg) in combination with AN2728 ointment (50 mg, 2%) or control solvent every 24 h. The weight and wound size of each mouse were recorded every 2 d until 10 d post infection.

The mice were euthanized (pentobarbital sodium, 30 mg/kg for intraperitoneal injection, then cervical dislocation) for bacterial loading analysis, histological observation and inflammatory response detection at 72 h postinfection. The infected skin tissue from the mice (*n* = 6 per group) was obtained to confirm the bacterial burden and was also stained with hematoxylin and eosin (H&E) for pathological analysis. In addition, inflammatory factors, including IL-1β, IL-6, TNF-*α* and IFN-*γ*, were detected in the livers of the mice (*n* = 3 per group) by ELISA kits (BioLegend, the United States) following the manufacturer’s instructions.

### Statistics and reproducibility

SPSS version 19.0 and GraphPad Prism 7.0 were used for statistical analysis, and the data are presented as the mean ± standard deviation. A t test was used to determine the significance of differences between two groups. Differences were considered statistically significant when *p* < 0.05.

## Results

### AN2728 synergized with multiple antibiotics against *Staphylococcus aureus*, especially MRSA

**Figure 1:**
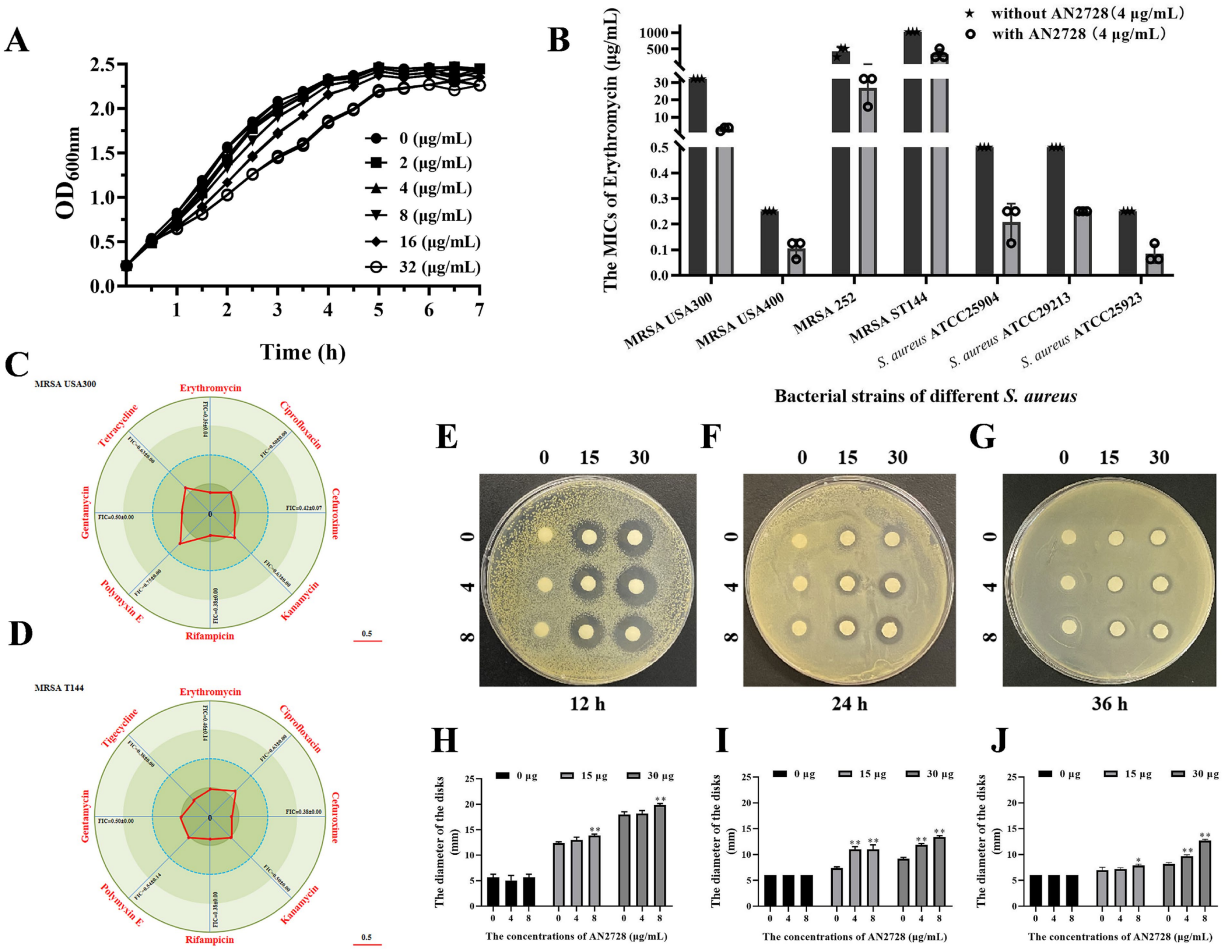
AN2728 restores the antibacterial activity of different antibiotics. **(A)** Growth curves for MRSA USA300. **(B)** AN2728 had a significant synergistic effect with erythromycin against bacterial strains of different *S. aureus* (*n* = 3). **(C,D)** Contribution capacity charts of FICI showing that AN2728 had synergistic effects with erythromycin, cefuroxime, rifampicin and gentamycin against MRSA USA300 and MRSA T144 (*n* = 3). **(E–J)** Confirmation of synergism by detecting the diameter of disks with different concentrations of AN2728 and erythromycin at different times. * indicates *p* < 0.05. ** indicates *p* < 0.01.

We applied the checkerboard MIC method to confirm that the conventional antibiotic AN2728 can have a synergistic antimicrobial effect. First, the results of the growth curve assay showed that AN2728 had no visible influence on the growth of MRSA USA300 at concentrations no greater than 32 μg/mL (Figure 1A). The results from the checkerboard MIC confirmed the synergistic effect between AN2728 and erythromycin, cefuroxime, rifampicin and gentamycin against MRSA USA300 and MRSA T144, and an additive effect was found between AN2728 and the other tested antibiotics (Figures 1C,D). Consistent with these results, AN2728 treatment effectively restored the sensitivity of *S. aureus* to erythromycin (Figure 1B). The bacteriostatic activity of AN2728 combined with erythromycin was measured by determining the diameter of the disks. As shown in Figures 1E–J, the size of the inhibition zones gradually increased in a dose-dependent manner with increasing concentrations of AN2728 and erythromycin.

**Figure 2:**
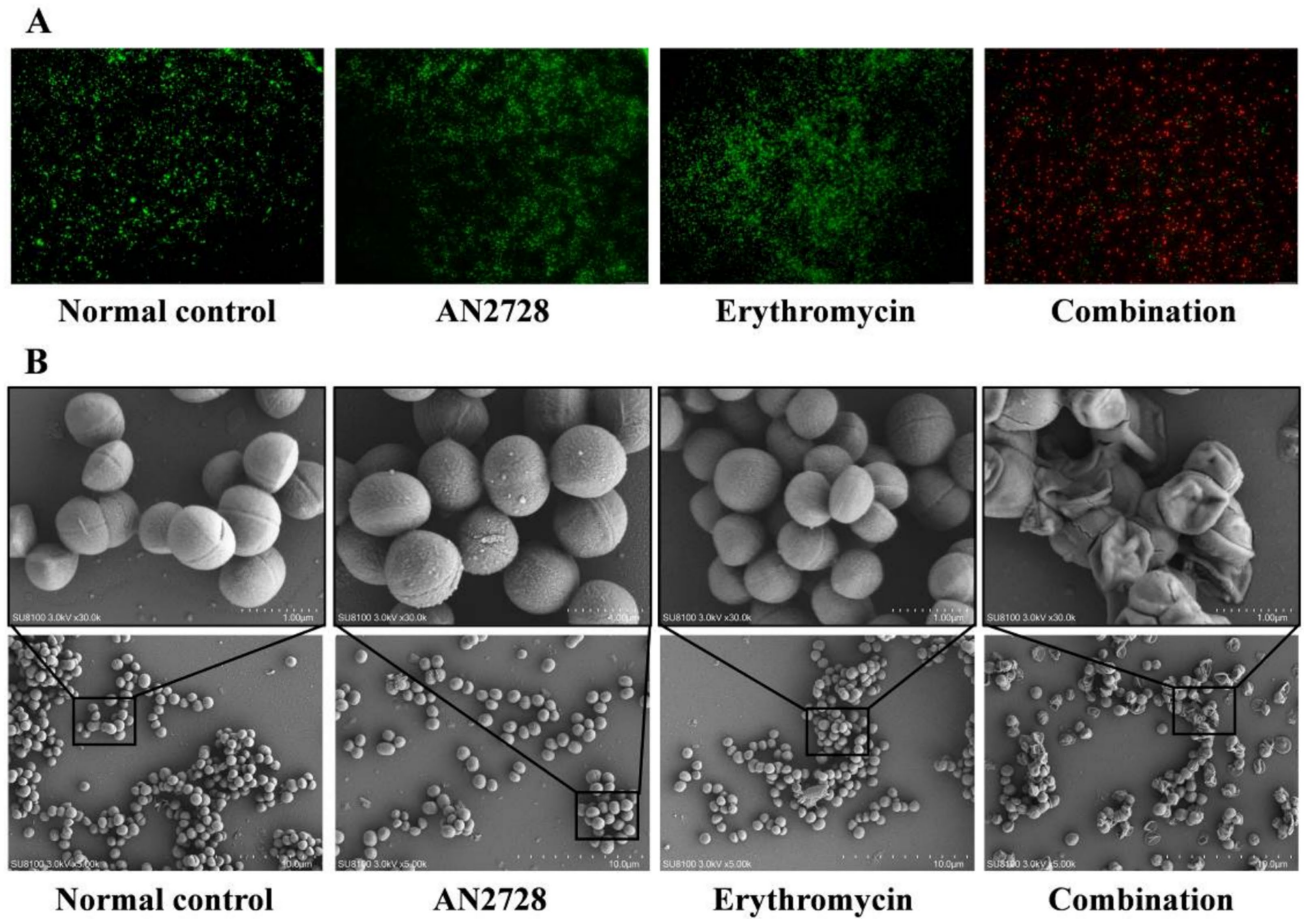
AN2728 combined with erythromycin effectively kills MRSA USA300. **(A)** Fluorescence labeling analysis for MRSA USA300 were treated with AN2728 (4 μg/mL), erythromycin (8 μg/mL) the combination or normal control (scale bar = 50 μm). **(B)** SEM analysis for MRSA USA300 was performed with the above treatment (SU8100 3.0 kV × 30.0 k/5.00 k, bar = 1.00 μM/10.0 μM). The experiments were repeated three times independently, and representative data are displayed.

**Figure 3:**
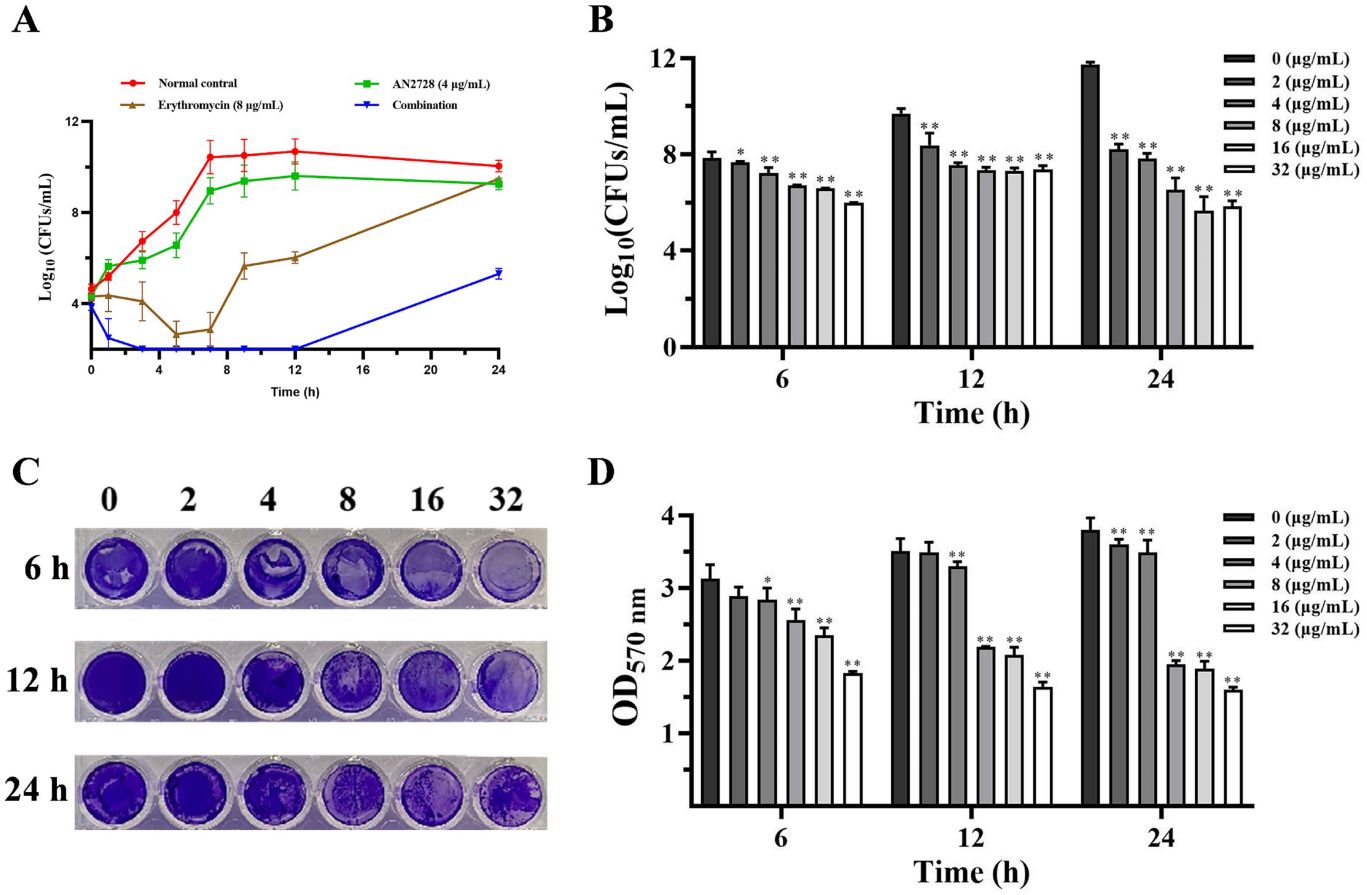
AN2728 alone inhibits the formation of MRSA biofilms. **(A)** Time-killing curves for AN2728, erythromycin, combination and control treatment against MRSA USA300. **(B)** Bacterial burden in the biofilm formation by MRSA USA300 treated with different concentrations of AN2728. **(C,D)** Quantification of biofilm biomass was detected by crystal violet (CV) staining with the absorbance at OD 570nm.Values represent the averages of three independent experiments. * indicates *p* < 0.05. ** indicates *p* < 0.01.

MRSA USA300 was further used to determine whether AN2728 combined with erythromycin had an efficient bactericidal effect *in vitro.* As shown in Figure 2A, more red-dyed bacteria (dead bacteria) were found only in the combination group, and the combination therapy led to visible shrinkage, rupture and invagination of the bacterial cell wall, increased cell permeability and thorough death of MRSA USA300 (Figure 2B). In agreement with these results, compared with both the monotherapies and the normal control, AN2728 in combination with erythromycin significantly killed all the tested bacteria in 24 h (Figure 3A). Taken together, our results established that AN2728 could effectively restore the bactericidal effect of erythromycin *in vitro*.

### AN2728 interfered with biofilm formation, membrane integrity and metabolism in MRSA

*Staphylococcus aureus* biofilm formation is the first step in establishing infection and persistence. Thus, we further examined the effect of AN2728 on the formation of bacterial biofilms. The number of bacteria in the biofilm and the quantified biofilm biomass were significantly decreased by AN2728 treatment in a dose-dependent manner at different times (Figures 3B–C).

**Figure 4:**
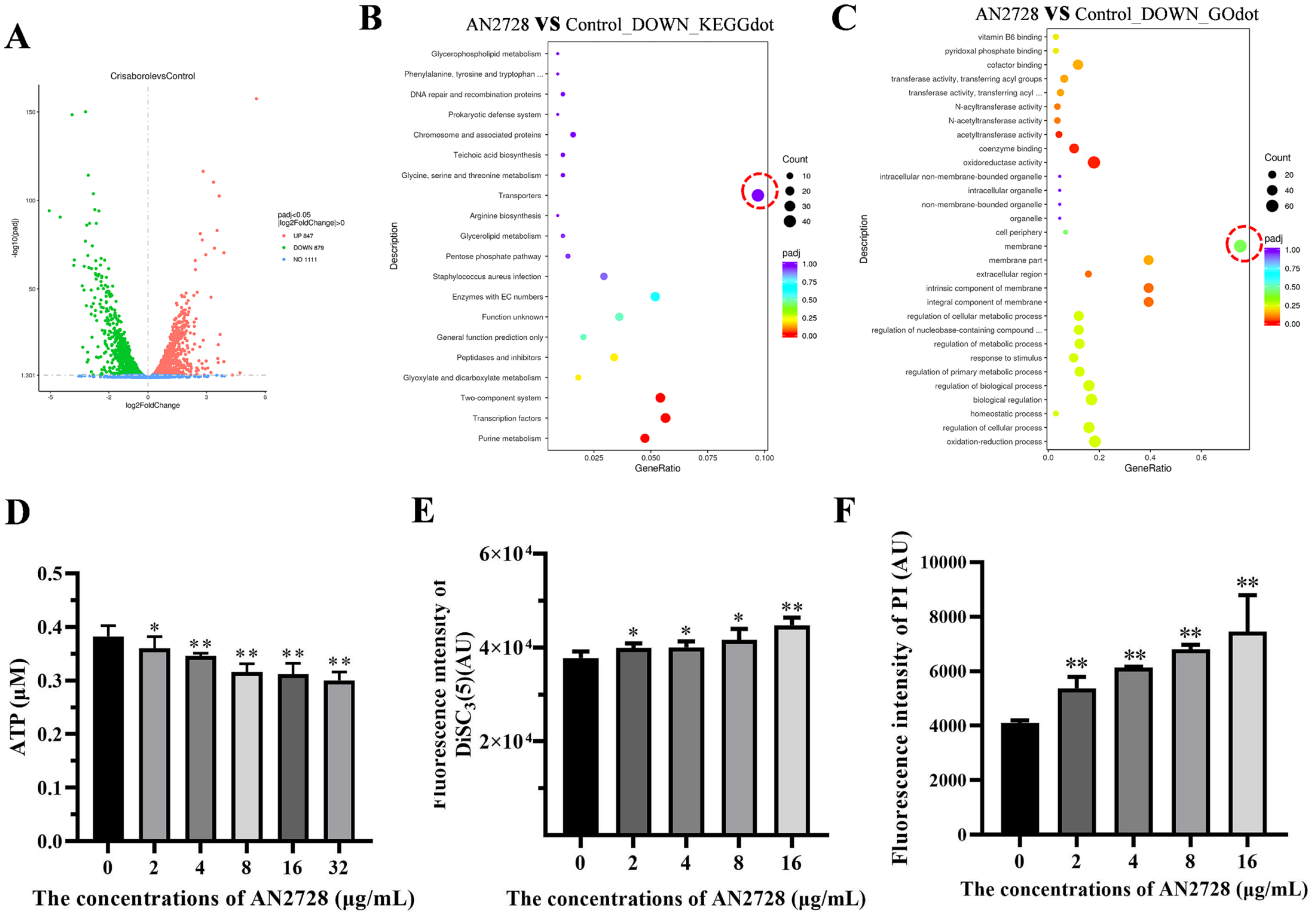
AN2728 reduces the function of MRSA membrane and the transporter capacity. **(A)** The differentially expressed genes of MRSA USA300 treated with AN2728 or normal control. **(B,C)** The downregulated genes were mainly focused on the transporters of MRSA USA300 by KEGG enrichment analysis, and GO enrichment analysis shows downregulated genes associated with MRSA membrane (outlined in redcircle). Further, the intracellular ATP levels **(D)**, membrane depolarization **(E)**, and the permeability of the inner membrane **(F)** of MRSA USA300 were also confirmed. The experiments were repeated three times independently. * indicates *p* < 0.05. ** indicates *p* < 0.01.

Transcriptome analysis was used to screen possible mechanisms of AN2728 in bacteria. Transcriptome analysis revealed that 879 genes were downregulated and 847 genes were upregulated in the 32 μg/mL AN2728 treatment group compared with the control group (Figure 4A). All the genes were associated with bacterial biological processes, cellular components and molecular functions. The transcriptome data were analyzed by pathway enrichment analysis (KEGGdot and GOdot), and the most significantly downregulated genes were associated with transporters and membranes (Figures 4B,C). This suggests that AN2728 may influence bacterial energy metabolism and membrane formation to exert combined antibacterial activity. As expected, AN2728 treatment significantly inhibited intracellular ATP generation in MRSA USA300 bacteria (Figure 4D). Moreover, AN2728 administration decreased the proton motive force, as evidenced by the disruption of IM integrity and the proton gradient (Figures 4E,F).

### Combination therapy of AN2728 and erythromycin synergistically alleviates damage to mice from MRSA skin infection

**Figure 5:**
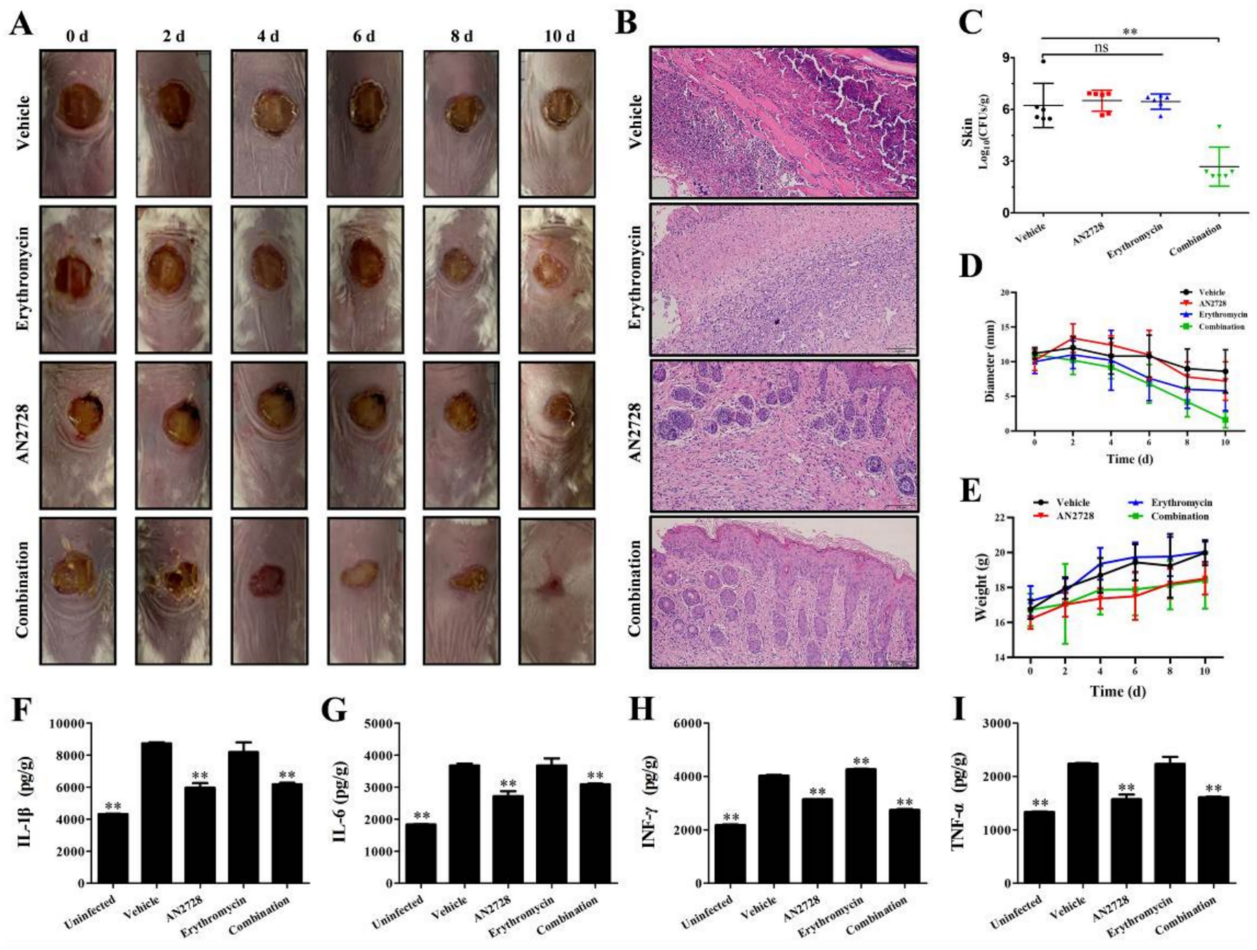
Effects of AN2728 and erythromycin combination therapy against MRSA USA300. Mouse skin infection model monitoring including the wound changes observed with the naked (All the photographs in each group came from a single mouse) **(A)**, histopathological observation **(B)**, bacterial loading **(C)** diameter of wound **(D)**, and weight variation **(E)**. Cytokines levels of IL-1β **(F)**, IL-6 **(G)**, IFN-*γ* **(H)** and TNF-*α* **(I)** in liver homogenates were detected using ELISA kits. * indicates *p* < 0.05. ** indicates *p* < 0.01.

The synergy between AN2728 and erythromycin was further confirmed by experimental therapeutics in a mouse model of skin infection. The size of the infected wounds on the skin of the mice gradually decreased at 12 d postinfection (Figures 5A,D), and only the wounds in the combination treatment group tended to close (Figure 5A). The combination treatment led to significant remission of pathological damage to the skin (Figure 5B). Additionally, a reduction in CFU counts of more than three orders of magnitude was observed in the wounds of the skin in the combination therapy group compared with the other groups (Figure 5C). Notably, there was no significant difference in body weight between the combination therapy group and the other groups (Figure 5D).

Erythromycin treatment notably reduced inflammation. However, AN2728 treatment and combination treatment significantly decreased the levels of the typical inflammatory-related factors IL-1β (Figure 5F), IL-6 (Figure 5G), IFN-*γ* (Figure 5H) and TNF-*α* (Figure 5I) in the liver. The results indicated that AN2728 exhibited a favorable anti-inflammatory effect, whereas erythromycin did not significantly alleviate inflammatory markers. Thus, our results indicate that AN2728 combined with erythromycin significantly alleviates damage caused by MRSA skin infection in mice.

## Discussion

MRSA is one of the most common causes of health care-associated pneumonia in hospitals, accounting for up to 40% of clinically relevant pneumonia cases (Rubinstein et al., 2008; Rose et al., 2015). MRSA strains are categorized as hospital-acquired MRSA (HA-MRSA), community-acquired/associated MRSA (CA-MRSA), or livestock-associated MRSA (LA-MRSA) according to the site of origin. HA-MRSA is currently the most commonly reported MRSA strain exhibiting low virulence and is a serious concern in nosocomial settings worldwide (Rose et al., 2015; Lacey et al., 2022). CA-MRSA is highly pathogenic compared to HA-MRSA, and its outbreaks and epidemics may be characterized by exposure within crowded public transportation, poorer public living habits, poor hygienic conditions and shared equipment/supplies (Mlynarczyk-Bonikowska et al., 2022). The typical LA-MRSA clone CC398 represents only a small fraction of all MRSA strains infecting humans and is almost exclusively limited by occupational exposure. However, it can be highly pathogenic (Aires-de-Sousa, 2017). Therefore, the first priority in trying to avoid further nosocomial outbreaks may be the need to control both community and animal reservoirs by adopting a One Health approach (Wang et al., 2023; Parisi et al., 2019).

In recent years, macrolide antimicrobials, such as erythromycin, roxithromycin, azithromycin, and clarithromycin, have been widely used in clinical practice (Hu et al., 2023). However, an increasing number of MRSA strains are resistant to erythromycin, and the rate of resistance of MRSA to erythromycin is as high as 80% in tertiary hospitals in China (Hu et al., 2018). The resistance of *S. aureus* to macrolide antimicrobials is often associated with members of the erythromycin ribosomal methylase (*erm*) gene family, such as the *ermA*, ermB, ermC, and *ermT* genes, which can be carried by plasmids generally located in conjugated or nonconjugated transposons (Wan et al., 2016). Thus, with severe MRSA resistance to erythromycin, topical ointment appears to be successful at controlling MRSA infections at a high effective concentration. In our study, the specific synergistic mechanism of AN2728 in combination with different antibiotics (especially erythromycin) needs to be further explored, while the exact mechanism of AN2728 targeting membrane-associated proteins in *S. aureus* also needs to be verified by more experiments.

Crisaborole targets PDE4 to degrade intracellular cyclic adenosine monophosphate, thereby suppressing the release of cytokines by affecting downstream regulation of activating T-cell signaling pathway-associated nuclear factors (Dastidar et al., 2007; Zane et al., 2016). This means that PDE4 inhibitors such as AN2728 have good therapeutic effects on psoriasis via clear mechanisms, but exactly how AN2728 combined with erythromycin affects purulent skin inflammation caused by *S. aureus* infection is unknown. Here, we confirm that AN2728 in combination with erythromycin achieves its therapeutic effects by exerting a direct *S. aureus*-killing effect and marked anti-inflammatory effect (Graphical abstract). There are a variety of antibiotics, such as erythromycin, that can be used topically, so our results provide a variety of viable drug combinations or strategies for treating MRSA skin infections (Tao, 2020; Lou et al., 2023; Khan et al., 2022). In addition, whether AN2728 is synergistic *in vivo* with different antibiotics that have been validated to have synergistic effects in this study needs to be further explored.

## Data Availability

The datasets presented in this study can be found in online repositories. The names of the repository/repositories and accession number(s) can be found at: https://www.ncbi.nlm.nih.gov/, PRJNA1092827.
